# Random intercept EFA of personality scales

**DOI:** 10.1016/j.jrp.2014.07.001

**Published:** 2014-12

**Authors:** Julian Aichholzer

**Affiliations:** Department of Methods in the Social Sciences, University of Vienna, Rathausstraße 19, 1010 Vienna, Austria

**Keywords:** Factor analysis, ESEM, Simple structure, Acquiescence, Big Five, NEO-FFI, BFI

## Abstract

Previous research suggests that simple structure CFAs of Big Five personality measures fail to accurately reflect the scale’s complex factorial structure, whereas EFAs generally perform better. Another strand of research suggests that acquiescence or uniform response bias masks the scale’s “true” factorial structure. Random Intercept EFA (RI-EFA) captures acquiescence as well as the complex item-factor structure typical for personality measures. It is applied to the NEO-FFI and the BFI scale to test whether an accurate model-to-data fit can be achieved and whether the “clarity” of the factorial structure improves. The results lend confidence in the general effectiveness of RI-EFA whenever acquiescence bias is an issue. Example M*plus* code is provided for replication.

## Highlights

• Random Intercept EFA (RI-EFA) adjusts for acquiescence bias in self-reports.
• RI-EFA was examined with regard to the NEO-FFI and BFI personality scales.
• RI-EFA provides better model-to-data fit than standard CFA or EFA.
• The clarity of Big Five factor structures improves when applying RI-EFA.
• RI-EFA can be extended to further analytical applications.

## 1 Introduction

The evaluation of the structure and validity of new and existing measures of psychological traits or attitudes is a central task in quantitative empirical research. One of the most important statistical techniques in this area is the common factor model and its variants, Exploratory Factor Analysis (EFA) and Confirmatory Factor Analysis (CFA). In particular, this study focuses on assessing the factorial structure of “Big Five” personality trait measures by means of factor analysis.

First, a relatively large body of literature has shown that the unrestricted EFA model is often better suited to reflect the complex factorial structure of Big Five scales than the more restrictive “simple structure” CFA approach (e.g. Asparouhov and Muthén, 2009, Booth and Hughes, in press, Borkenau and Ostendorf, 1990, Hopwood and Donnellan, 2010, Marsh et al., 2010). This is mainly because CFA, or more precisely, its notion of independent clusters of items assumes that indicators perfectly measure only one target trait, though this condition is rarely met.

Second, the literature shows that self-report measures are commonly plagued by different sorts of method bias (see, for an overview, Podsakoff, MacKenzie, & Podsakoff, 2012). Particularly, acquiescence response style, also known as directional or uniform response bias (hereafter ARS), has been identified as one of the most important nuisance factors in personality measurement (e.g. McCrae et al., 2001, Rammstedt and Farmer, 2013). It therefore has become standard to mitigate this problem by using semantically balanced scales in questionnaires, i.e., using positively (pro-trait) and negatively (con-trait) worded items.

Most importantly, ARS can distort the factorial structure, i.e., convergent and discriminant validity of the questionnaire items as systematic measurement error is introduced (Podsakoff et al., 2012). As a consequence, the “true” factorial structure, especially in multidimensional instruments (e.g. Big Five scales), is misrepresented. Several approaches have therefore tried to remedy this problem by means of adjustment techniques that mitigate the impact of ARS, for instance, by using a CFA model of ARS (Billiet and McClendon, 2000, Maydeu-Olivares and Coffman, 2006) or by subtracting the within-person mean response from each item, also called ipsatization (Rammstedt & Farmer, 2013).

## 2 Aim and contribution of this study

In this study I investigate the properties of Random Intercept EFA (RI-EFA) (Aichholzer, in preparation). RI-EFA treats ARS as an individual random intercept (RI) factor extending the standard EFA model. In particular, the RI-EFA approach is applicable whenever multidimensional and semantically balanced scales of psychological constructs or attitudes are examined. This research demonstrates the usefulness of RI-EFA using two standard Big Five personality scales, the German versions of the NEO-FFI (Borkenau & Ostendorf, 1993) and the BFI (Rammstedt & John, 2005). In addition, I test four hypotheses that have been put forward in previous research:

• H_1_: Measurement models with complex item-factor structure (EFA) commonly fit better to Big Five data than restricted simple structure models (CFA) (e.g. Hopwood and Donnellan, 2010, Marsh et al., 2010).
• H_2_: Measurement models with adjustment for ARS fit the data better than models without such adjustment (e.g. Billiet and McClendon, 2000, Maydeu-Olivares and Coffman, 2006).
• H_3_: ARS adjustment improves the clarity of factor loading patterns in ways that approach the ideal “perfect simple structure” form (e.g. McCrae et al., 2001, Rammstedt and Farmer, 2013).
• H_4_: ARS adjustment improves the clarity of factor loading patterns in particular in populations that are prone to ARS (e.g. Rammstedt and Farmer, 2013, Rammstedt et al., 2010).

H_1_ is tested by comparing common model fit criteria of standard CFA with EFA. H_2_ is tested by comparing RI-EFA with the other models. H_3_ and H_4_ are assessed by comparing empirical factor loading structures with a perfect simple structure matrix (i.e., perfect −1/0/+1 entries). The contribution of this study is thus twofold: First, a novel method in the field of factor analysis is tested with regard to its applicability to established scales. Second, these scales are re-evaluated using this new method.

## 3 The RI-EFA model

Recently, Aichholzer (in preparation) has presented the RI-EFA model that allows researchers to combine standard EFA, i.e., an unrestricted or complex item-factor loading matrix, with a random intercept (RI) factor that represents ARS or uniform response bias more generally. So far, the latter option was only available for restricted CFA models (Billiet and McClendon, 2000, Maydeu-Olivares and Coffman, 2006).

The RI-EFA model is an extension of the common factor model and reads as follows

$$Y_{\mathrm{ki}}=\tau_{k}+\Lambda^{*}\cdot \eta_{\mathrm{ji}}+1\cdot \alpha_{i}+\varepsilon_{\mathrm{ki}}$$

where the *k* × 1 vector of observed scores *Y_ki_* for a respondent *i* is derived from item intercepts *τ_k_*, unconstrained loadings *λ_kj_* on a hypothesized number of *j* latent factor scores *η_ji_* that are contained in the *k* *×* *j* factor loading matrix *Λ*, the individual RI/ARS factor *α_i_*, and a vector of all unique (residual) factors *ε_ki_*. Note that the factor loading matrix *Λ* has to be rotated by some criterion (i.e., by orthogonal or oblique rotation) for the model to be identified (Λ^*^ after rotation). Common assumptions in factor analysis apply, namely that factors are uncorrelated with residuals and residuals are mutually uncorrelated. Regardless of the rotation method used for the latent “substantial” factors, *α* is assumed to be orthogonal to factors and item uniquenesses for identification, i.e.:

$$\mathrm{Cov}(\eta_{j}\text{,}\varepsilon_{k})=\mathrm{Cov}(\varepsilon_{k}\text{,}\varepsilon_{k^{'}})=0\;\text{for}\;\varepsilon_{k}\;\neq \;\varepsilon_{k^{'}}\;\;\text{and}\;\mathrm{Cov}(\eta_{j}\text{,}\alpha )=\mathrm{Cov}(\varepsilon_{k}\text{,}\alpha )=0$$

Further note that the factor *α* has a constant loading vector of **1** on all indicators, regardless of their keying (pro-trait or con-trait), while its variance is freely estimated and tested to be non-zero.^1^ The RI-EFA model is thus a hybrid model that combines an EFA part where item-factor loadings are freely estimated and a restricted CFA part where item-factor loadings on the RI/ARS factor *α* are restricted to follow a predefined pattern. Due to its specific factor loading structure *α* must not be confused with other factors of personality that load on all items (see Anusic, Schimmack, Pinkus, & Lockwood, 2009). In other words, *α_i_* represents a uniform shift of individual item responses independent from substantial factors. Hence, it can be considered an issue of differential item functioning (DIF) or violation of measurement invariance.

Furthermore, the model can be estimated quite easily applying the Exploratory Structural Equation Modeling (ESEM) framework (Asparouhov & Muthén, 2009) and the M*plus* software (Muthén & Muthén, 1998–2012) (see the Appendix A for example M*plus* code).

## 4 Materials and methods

### 4.1 Instruments

This study investigates the German 60-item NEO-FFI (Borkenau & Ostendorf, 1993) and the German 44-item BFI (Rammstedt & John, 2005). Both the NEO-FFI and the BFI are based on the Big Five taxonomy of Extraversion (E), Agreeableness (A), Conscientiousness (C), Neuroticism (N), and Openness to experience (O). All items were measured on a 5-point scale with endpoints labeled as 1 – *strongly disagree* to 5 – *strongly agree* in the NEO-FFI and 1 – *does not apply at all* to 5 – *applies completely* for self-reports in the BFI. In the sample used here, the Cronbach’s Alpha estimates for the hypothesized Big Five dimensions were .75 (E), .72 (A), .84 (C), .82 (N), and .66 (O) for the NEO-FFI and .81 (E), .76 (A), .80 (C), .74 (N), and .82 (O) for the BFI. Note that previous research has already investigated the factorial structure of these instruments using different factor analytic strategies (Booth and Hughes, in press, Marsh et al., 2010, Rammstedt and Farmer, 2013).

### 4.2 Data

The NEO-FFI and the BFI were administered in a random sample of the German population (aged 18 and above) as part of a larger study on personality and political behavior (Schumann, 2004).^2^ The total sample with valid demographic data comprised *n* = 2508 respondents. Age ranged between 18 and 92 (*Mean* = 49, *SD* = 17) and 52% of the respondents were female, 48% male. The NEO-FFI was administered face-to-face, while the BFI was administered using self-administration as a drop-off (with *n* = 1492 participants in total).

### 4.3 Analysis

Analyses were conducted with standard simple structure CFA, EFA (or simple ESEM), and RI-EFA using linear MLR (maximum-likelihood with robust standard errors) estimation as well as WLSMV (weighted least square mean- and variance-adjusted) estimation for ordered categorical measures in M*plus* 7 (Muthén & Muthén, 1998–2012). Global goodness-of-fit indices were inspected for each model in addition to the *χ*^2^-test. The reason for this is that *χ*^2^-tests commonly result in a rejection of the model when applied to large samples. According to common fit criteria (see Marsh, Hau, & Wen, 2004), acceptable fit is achieved when CFI > .90, TLI > .90, and RMSEA < .08, whereas excellent fit is achieved when CFI > .95, TLI > .95, and RMSEA < .05. Further, better fit of a model is also supported by lower BIC values.

For all empirical analyses the theoretical five-factor structure was imposed to investigate the hypothesized structure of the NEO-FFI and the BFI. Solutions for the rotated factor loading matrix in the EFA part were computed applying oblique Quartimin rotation (for other factor rotation criteria see Asparouhov & Muthén, 2009).

## 5 Results

I start by comparing the fit measures for the different modeling strategies (Table 1). First, the results reconfirm previous evidence suggesting that unrestricted EFA (or simple ESEM) fits Big Five data considerably better than standard simple structure CFA (see Table 1), which supports H_1_.

**Table 1:** Summary of goodness-of-fit indices for different modeling approaches.

Measure	Method	Model	*χ*^2^	d.f.	*p*(*χ*^2^)	CFI	TLI	RMSEA	BIC
NEO-FFI (*n* = 2411)		CFA	12,526	1,700	<.01	.661	.647	.051	392,714
	MLR	EFA	4,716	1,480	<.01	.899	.879	.030	385,128
		RI-EFA	4,273	1,479	<.01	.913	.895	.028	384,607
		CFA	24,644	1,700	<.01	.683	.670	.075	n.a.
	WLSMV	EFA	5,907	1,480	<.01	.939	.927	.035	n.a.
		RI-EFA	5,078	1,479	<.01	.950	.940	.032	n.a.

BFI (*n* = 1428)		CFA	7,074	892	<.01	.640	.618	.070	165,411
	MLR	EFA	2,404	736	<.01	.903	.875	.040	160,651
		RI-EFA	1,787	735	<.01	.939	.921	.032	159,896
		CFA	13,807	892	<.01	.656	.635	.101	n.a.
	WLSMV	EFA	3,470	736	<.01	.927	.906	.051	n.a.
		RI-EFA	2,302	735	<.01	.958	.946	.039	n.a.

*Note:* A five-factor solution was hypothesized in all cases. EFA solutions use Quartimin rotation. All models converged. n.a. = not available. No post-stratification weights were applied. Sample size *n* refers to listwise deletion.

Second, having established that EFA fits better than CFA, it is shown that RI-EFA has a better model-to-data fit than EFA for the NEO-FFI and the BFI, which supports H_2_. Better fit for RI-EFA vs. EFA is further supported by lower BIC values, significant *χ*^2^ reduction (each *p* < .01 at −1 degrees of freedom), and rejection of Var(α)=0 (at *p* < .01). If the RI/ARS bias was irrelevant, the model fit should be very similar or equal. Note that these findings hold regardless of the estimation method (MLR or WLSMV).

Third, as suggested by Hopwood and Donnellan (2010), I compared the congruence of the empirical factor loading pattern to a perfect simple structure matrix by assigning each item to its hypothesized factor, using Tucker’s congruence coefficient *c* (see Table 2, detailed factor loading results using MLR estimation are available as supplemental materials). After partialling out the additional RI/ARS factor by means of RI-EFA, there is convincing evidence in support of further alignment (*Δ* of congruence) of items towards the hypothesized five-factor personality structure, which confirms H_3_. In other words, the matrix congruence to perfect simple structure is always larger for the RI-EFA factor loading patterns. Further, the variance explained by the RI/ARS factor can be computed using squared standardized loadings, 2.8% (NEO-FFI) and 7.5% (BFI) with MLR estimation, which is largely similar to previous findings (3–4%) (e.g. Anusic et al., 2009, Billiet and McClendon, 2000).

**Table 2:** Estimates of factor loading matrix congruence to perfect simple structure.

Measure	Model	Total sample	Low/high education
NEO-FFI	EFA	.78	.73/.77
	RI-EFA	.84	.78/.83
	*Δ*	.06	.05/.06
	*n*	2411	1095/491

BFI	EFA	.73	.71/.81
	RI-EFA	.90	.86/.90
	*Δ*	.17	.15/.09
	*n*	1428	677/290

*Note:* A five-factor solution was hypothesized in all cases. Entries indicate congruence coefficient *c* for the MLR Quartimin rotated solution. Sample size *n* refers to listwise deletion.

Fourth, I look at subpopulations with potentially different amounts or unequal variance in ARS. A common proxy is the respondent’s educational level (Rammstedt et al., 2010) which was operationalized by contrasting “low” education (lower secondary education or less) vs. “high” education (admission to tertiary education or completed university degree), whereas “medium” education was omitted. The results reconfirm that the factor loading congruence to a hypothesized five-factor simple structure is, in general, lower among less educated respondents. Applying RI-EFA improves the clarity of factor loading patterns among both groups, though it improves especially among less educated respondents who filled out the BFI, which partly supports H_4_.

In sum, the results suggest that applying RI-EFA makes a greater difference for revealing the “true” structure in the case of the BFI, whereas the impact of response bias on substantial results seems to be smaller for the NEO-FFI. However, it should be mentioned that differences between the two instruments may also reflect the use of different survey modes (F2F vs. P&P).

## 6 Discussion

This research shows that RI-EFA can be used as an effective factor-analytic method. It provides a useful tool for researchers when they would like to investigate the factorial structure of multidimensional scales based on semantically balanced or heterogeneous items. The results support the improvement of model-to-data fit and suggest that the hypothesized factor structure became “clearer” (i.e., it approximated simple structure). Moreover, given that the identified bias resembles ARS, it may be the preferred method whenever heterogeneous populations with potentially different response behavior are studied or when specific measurement conditions apply that evoke such response bias.

The main advantage which makes RI-EFA superior to other approaches, such as Principal Component Analysis (PCA) of ipsatized data (Rammstedt & Farmer, 2013), is that RI-EFA fits into the larger family of common factor models and structural equation modeling or ESEM (Aichholzer, in preparation). Therefore RI-EFA can be extended to testing measurement invariance over subgroups or over time as well as to testing covariates of the RI/ARS factor and, hence, causes of such bias. As has been shown, RI-EFA can also be extended to the case of ordered categorical indicators but also to binary items using WLSMV estimation (Asparouhov & Muthén, 2009).

Nevertheless, there are some limitations to RI-EFA, some of which apply to the ESEM framework more generally (e.g. Booth and Hughes, in press, Herrmann and Pfister, 2013, Marsh et al., 2010). Firstly, RI-EFA is neither a strictly exploratory nor a strictly confirmatory method, but it might be valuable as a “first-step” method in the sense that it is less rigorous compared to the CFA approach. Second, different rotation criteria (Quartimin, Geomin, Varimax, etc.) should be examined as factor loadings and between-trait correlations are dependent on the rotation criterion used. Third, it is still a matter of debate to what extent the factor scores and associations with covariates will differ between the different approaches, i.e., CFA, EFA/ESEM, or RI-EFA (also see Booth and Hughes, in press, Herrmann and Pfister, 2013). Apart from that, quite often the items in a scale are not fully but partially balanced (e.g. NEO-FFI and BFI), that is, some subscales use an even number of pro-trait and con-trait items, whereas others are primarily keyed in one direction. Indeed, the ultimate goal of the RI/ARS factor is that it conveys all information from balanced subsets of items to also control for ARS in other items and subscales that are partly or completely unbalanced. One could also let a selection of balanced item sets define the RI/ARS factor (for a set of items in the BFI see Rammstedt & Farmer, 2013), whereas loadings are set to +1, and freely estimate loadings of the remaining items on that factor. Such a model can then be compared to the basic RI-EFA model (constant loadings of +1). Still, as previously noted (Aichholzer, in preparation), the construct validity of the RI/ARS factor itself will be higher with an increasing number of balanced items that come from heterogeneous constructs. However, less is known about the amount of balancing in scales in order to fully identify and accurately address the type of bias which is modeled here. Future studies might investigate these issues in more depth.

## Figures and Tables

**Table:** 

Variable:
NAMES = item1-item60; ! Example for 60-item NEO-FFI
!CATEGORICAL = item1-item60; ! optional
Analysis:
ESTIMATOR = MLR; ! Choose estimator: MLR, WLSMV, etc.
ROTATION = QUARTIMIN; ! Select rotation criterion
Model:
F1-F5 BY item1-item60 (*L); ! Define 5-factor Lambda matrix
RI BY item1-item60@1; ! Define RI/ARS factor loadings
RI WITH F1-F5@0; ! RI/ARS is orthogonal
Output:
STDYX; ! Compute fully standardized factor loadings

## Footnotes

1 Note that other approaches also follow the assumption of a uniform bias, for instance, when a constant is subtracted from all individual item responses (ipsatization).

2 Note that the item ordering differs from the original NEO-FFI. Item_1 and Item_21 were interchanged.
